# Supporting implementation through collaborative learning: facilitating access to clinical expertise

**DOI:** 10.1186/1748-5908-10-S1-A25

**Published:** 2015-08-20

**Authors:** Alicia C Bunger, Nathan J Doogan, Rochelle F Hanson

**Affiliations:** 1 College of Social Work, Ohio State University, Columbus, OH 43210, USA; 2 College of Public Health, Ohio State University, Columbus, OH 43210, USA; 3 National Crime Victims Research and Treatment Center, Department of Psychiatry & Behavioral Sciences, Medical University of South Carolina, Charleston, SC 29425, USA

## Objective

Learning collaboratives (LCs) have potential to promote implementation of empirically supported treatments (ESTs) in healthcare organizations and systems by building networks among clinicians and with key content experts that facilitate shared expertise. However, the effectiveness of LCs for facilitating clinicians' access to expertise, and implementation success is untested. This study examines LCs' impact on how clinicians choose colleagues for professional advice by assessing the influence of expertise and experience on the evolution of advice-seeking patterns among LC participants.

## Methods

We examined advice-seeking among 146 LC participants (including 5 experts) from 27 agencies participating in a naturally occurring, regional scale-up of a mental health EST. Surveys were administered in-person at the first and last LC learning sessions (10 months apart). Participants nominated up to five individuals from whom they seek professional advice. A stochastic actor-oriented model of network dynamics modelled the maintenance, formation and dissolution of relationships among participants based on their expertise, EST experience, implementation team role, agency membership, and other features.

## Findings

Over time, participants maintained and built ties with faculty experts, supervisors, and colleagues from their own agency. Also participants reciprocated advice-seeking and connected colleagues to one another suggesting that advice-seeking became increasingly intensive, clustered and hierarchical with faculty experts positioned at the top. However, senior agency leaders were less likely to seek advice than clinicians.

## Impact

Given the importance of accessible clinical expertise and ongoing supervision for delivering ESTs with fidelity, LCs may support implementation by building clinicians' relationships with faculty experts and strengthening within-agency relationships with supervisors. Future research should test strategies that promote inter-agency ties among the senior leaders given the importance of external ties and senior leadership for successful EST implementation and sustainment.

## Funding

NIMH (R25-MH080916-01A2, T32-MH019117), VA (QUERI).

